# Microbial Community Responses to Increased Water and Organic Matter in the Arid Soils of the McMurdo Dry Valleys, Antarctica

**DOI:** 10.3389/fmicb.2016.01040

**Published:** 2016-07-18

**Authors:** Heather N. Buelow, Ara S. Winter, David J. Van Horn, John E. Barrett, Michael N. Gooseff, Egbert Schwartz, Cristina D. Takacs-Vesbach

**Affiliations:** ^1^Department of Biology, University of New MexicoAlbuquerque, NM, USA; ^2^Department of Biological Sciences, Virginia TechBlacksburg, VA, USA; ^3^Department of Civil, Architectural, and Environmental Engineering, Institute of Arctic and Alpine Research, University of Colorado BoulderBoulder, CO, USA; ^4^Department of Biological Sciences, Northern Arizona UniversityFlagstaff, AZ, USA

**Keywords:** metatranscriptomics, microbial ecology, Antarctica, soils in hyper-arid regions, amendments

## Abstract

The soils of the McMurdo Dry Valleys, Antarctica are an extreme polar desert, inhabited exclusively by microscopic taxa. This region is on the threshold of anticipated climate change, with glacial melt, permafrost thaw, and the melting of massive buried ice increasing liquid water availability and mobilizing soil nutrients. Experimental water and organic matter (OM) amendments were applied to investigate how these climate change effects may impact the soil communities. To identify active taxa and their functions, total community RNA transcripts were sequenced and annotated, and amended soils were compared with unamended control soils using differential abundance and expression analyses. Overall, taxonomic diversity declined with amendments of water and OM. The domain *Bacteria* increased with both amendments while *Eukaryota* declined from 38% of all taxa in control soils to 8 and 11% in water and OM amended soils, respectively. Among bacterial phyla, *Actinobacteria* (59%) dominated water-amended soils and *Firmicutes* (45%) dominated OM amended soils. Three bacterial phyla (*Actinobacteria*, *Proteobacteria*, and *Firmicutes*) were primarily responsible for the observed positive functional responses, while eukaryotic taxa experienced the majority (27 of 34) of significant transcript losses. These results indicated that as climate changes in this region, a replacement of endemic taxa adapted to dry, oligotrophic conditions by generalist, copiotrophic taxa is likely.

## Introduction

Microbial species in extreme environments are highly adapted to persist in their niches (Takacs-Vesbach et al., 2013). The specialization of taxa, combined with the relatively low taxonomic richness typically present in extreme environments (Neilson et al., 2012), make these microbial communities susceptible to environmental change (Tilman, 1996; Van Horn et al., 2014). The McMurdo Dry Valleys (MDV) of Antarctica are an extreme polar desert environment, characterized by freezing temperatures (Doran et al., 2002), low soil moisture, low soil organic matter (OM; Burkins et al., 2001), and the absence of higher plants and animals. While the climate of this region has been relatively stable for millennia (Denton et al., 1993), the MDV are currently on the threshold of climate induced change (Fountain et al., 2014), and impacts of these changes are already affecting dry-adapted soil species (Barrett et al., 2008a,b; Knox et al., 2016). In the past decade, increased solar radiation and decreased albedo has accelerated glacial melt, permafrost thaw, and the melting of massive buried ice, thereby increasing soil moisture and nutrient mobilization (Fountain et al., 2014). The study presented here uses experimental water and OM amendments to examine how soil communities in this extreme ecosystem will respond to the expected effects of climate change.

Soils are the largest habitat within the MDV and taxonomic studies have characterized the associated microbial communities revealing extensive bacterial richness (Lee et al., 2012; Van Horn et al., 2013, 2014) but limited eukaryotic diversity, which include nematodes, fungi, rotifers, and tardigrades (Freckman and Virginia, 1997, 1998; Adams et al., 2006). Detecting and distinguishing active taxa in these soils presents challenges. Soil respiration rates, a measure of biological activity in soils, have been shown to be sensitive to temperature (Parsons et al., 2004; Ball et al., 2009; but see Shanhun et al., 2012). Taxonomic shifts in response to water, resource amendments, and changing conditions have been identified using 16S rRNA gene sequencing (Tiao et al., 2012; Schwartz et al., 2014; Van Horn et al., 2014), however, these DNA-based studies cannot distinguish active taxa from dormant or non-viable cells. Non-viable cells are of particular concern in MDV soils, where freezing temperatures can delay degradation. Recently, stable isotope probing with H_2_^18^O water provided a first look at the taxonomy of definitively active bacteria in MDV soils and revealed a somewhat different community profile than prior 16S rRNA gene studies (Schwartz et al., 2014). The present study uses RNA sequencing as another means of distinguishing the active members of the soil community.

Beyond taxonomy, the next step in understanding this soil ecosystem is to examine the functions of active taxa. Components of MDV soil microbial activities have been investigated using various measures, including CO_2_ flux and other gas emissions (Burkins et al., 2001; Gregorich et al., 2006; Ball et al., 2009; Van Horn et al., 2014), extracellular enzyme assays (Zeglin et al., 2009; Van Horn et al., 2014), mineralization rate calculations (Barrett et al., 2005; Hopkins et al., 2006b), natural isotopic signature analysis of soil nutrients (Bao et al., 2000; Burkins et al., 2000; Barrett et al., 2006), and stable-isotope tracer techniques (Barrett et al., 2008b). Together these investigations have revealed an active and responsive community, but the functional profile of MDV soils has not been characterized. The goal of this study was to use total community RNA transcripts to characterize active soil taxa and their functions, and further, to determine their response to experimental soil amendments designed to simulate the expected effects of climate change in this region.

## Materials and Methods

### Site Description

**FIGURE 1:**
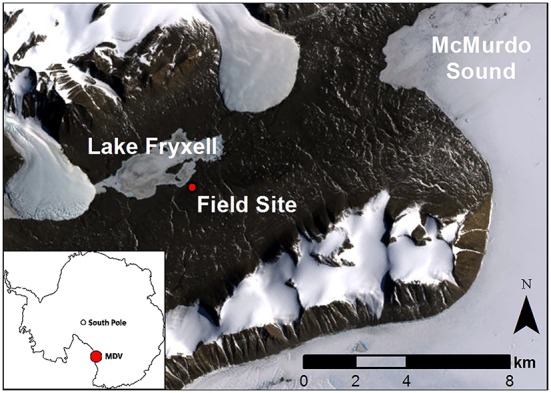
**Landsat image of the sampling location and Lake Fryxell within the McMurdo Dry Valleys**.

Field experiments took place in dry soils of the Taylor Valley, near the southern shore of Lake Fryxell (77°37′S, 163°12–13′E; **Figure 1**). Soil surface temperatures average -18.4°C annually (range -52.0 to 22.7°C), and the Fryxell lake basin experiences an average of 25.5 days above freezing per year (Doran et al., 2002). Annual precipitation in the basin is 20–37 mm (Fountain et al., 2010), but sublimation rates are high relative to precipitation inputs (Clow et al., 1988) and the proportion of snowfall that contributes to soil water content remains unknown (Eveland et al., 2013). Liquid water inputs are limited in time and space, occurring during the brief austral summer and concentrated in stream channels and wetted margins of glaciers, streams, and lakes. OM inputs are also limited, with soils averaging 0.03% organic carbon (Burkins et al., 2001). Although relic OM from Glacial Lake Washburn is present in these low elevation soils, most contemporary organic inputs are produced during the austral summer when algal mats proliferate in intermittently wet soils and aquatic habitats and are wind dispersed (Hopkins et al., 2006a; Moorhead, 2007) and *in situ* production by soil, endolithic, and hypolithic taxa occurs (Friedmann et al., 1993; Moorhead et al., 1999; Cary et al., 2010; Geyer et al., 2013).

### Sampling and Treatments

The sampling for this experiment was conducted as described previously by Van Horn et al. (2014) for the low salinity treatments of that study. On November 23, 2010, soils were collected to a depth of ∼12 cm using sterile scoops. Soils from this plot have electrical conductivity (a proxy for salinity) of 105 ± 4 μS, a pH of 9.0 ± 0.1, and contain 0.006 ± 0.002 percent nitrogen, and 0.027 ± 0.006 percent organic carbon (Van Horn et al., 2014). Soils were homogenized and placed into sterile cylindrical mesocosms (10 cm diameter × 17 cm deep), which were positioned in the excavated holes such that soil levels within the mesocosms matched the surrounding undisturbed soil level. Although the sampling, homogenization, and mesocosm filling represent disturbance events to the natural soil community, MDV regulations require that all treatments be contained. Therefore, control samples underwent the same sampling and containment processes despite no treatment addition to better assess what community responses resulting from treatments rather than disturbance.

One of three treatments, control, water addition, and OM amendment, was randomly assigned to each of the six mesocosms used for this study, yielding two samples per treatment. Treatments were applied following the methods of Van Horn et al. (2014): prior to treatment additions, soil moisture was measured using a HydroSense soil moisture probe calibrated for the soil in the experimental plots (Campbell Scientific). Sterile deionized water was added to water treatment mesocosms to achieve 10% soil water content by weight. OM was also added to achieve 10% soil water content by weight, which increased organic carbon by 0.005%, roughly one-tenth of the total soil organic carbon. The OM addition was a leachate prepared from native cyanobacterial mat collected from Lake Fryxell. The mat was steeped in sterile DI water and the resulting leachate (2,800 mg/liter dissolved organic carbon) was filter sterilized using 0.2 μm filters. For both treatment additions sterile syringes were used to penetrate the soil in five places within the mesocosm to a depth of 10 cm, distributing the water and/or OM throughout the depth. Soils were treated five times throughout the 30-day incubation period to maintain ∼10% soil moisture by weight. At the conclusion of the incubation period, soils were collected into sterile conical tubes and preserved with an equal volume of sucrose lysis buffer (Giovannoni et al., 1990) and immediately stored at -20°C until processed further.

### Nucleic Acid Extraction and Sequencing

A minimum of 15 g soil was extracted from each sample (range = 15–30 g) to yield a minimum of 8 μg RNA per sample for sequencing. Total nucleic acid was extracted from the samples following the cetyltrimethylammonium bromide method of Mitchell and Takacs-Vesbach (2008). To retain only RNA, DNase I and accompanying buffer (Invitrogen) was added to the samples following the manufacturer’s protocol. Sequencing libraries were prepared using Illumina’s mRNA-seq kit following the manufacturer’s protocols, and sequencing was conducted on an Illumina HiSeq 2 × 100.

### Data Analysis

Fastq sequence files were uploaded to MG-RAST (Meyer et al., 2008) using the pipeline options: unassembled, allowing for replicates, human sequences screened out, and filtered with dynamic trimming settings (minimum phred score = 5). These fastq sequence files are publicly available on MG-RAST under project 12330^1^ with MG-RAST ID numbers 4614919.3, 4617349.3, 4617350.3, 4617351.3, 4620847.3, and 4620848.3.

Sequences passing MG-RAST pipeline quality controls were then annotated using the M5NR (Wilke et al., 2012) and SEED Subsystems (Overbeek et al., 2005) databases for taxonomic and functional analyses, respectively. Notably, the M5NR database annotates protein-coding reads, while previous taxonomic studies of these soil communities used 16S rRNA gene sequences and rRNA annotation databases (Schwartz et al., 2014; Van Horn et al., 2014). All annotations were made following the MG-RAST default settings of *e*-value ≤1*e -* 5, identity ≥60%, and alignment length ≥15 base pairs.

After annotation, biological replicates were pooled by treatment, and analyzed in R (R Development Core Team, 2011), using the phyloseq (McMurdie and Holmes, 2013) and DESeq2 (Love et al., 2014) packages. Differences in the abundance of taxa and the variance in expression of transcripts were characterized using the DESeq2 parameters fitType = “local” and an adjusted *p*-value threshold of 0.05 to calculate log_2_ fold changes between treatments. Taxonomy was assigned to transcripts of significantly over-expressed functions. For functions that were under-expressed in water or OM amended samples, taxonomy was assigned to the transcripts of those functions in the control samples because in some cases the transcripts were not present in amendment samples. Similarity percentage analysis (SIMPER), performed in Community Analysis Package 5, was used to determine the OTUs that contributed most to the observed dissimilarity between treatments.

Secondary metabolite analysis of the control soils was performed using HMMER v3.1b2 (Finn et al., 2011) with a cutoff bit score of 20. Python scripts and workflow provided by the FOAM database (Prestat et al., 2014) were used to count the best hits from the HMMER run. The following Pfam database (Finn et al., 2013) HMMER profiles were used: ABM (PF03992), Actino_peptide (PF14408), Acyl_transf_1 (PF00698), Acyltransferase (PF01553), Antimicrobial18 (PF08130), Bacteroid_pep (PF14406), Carbam_trans_C (PF16861), Condensation (PF00668), FAE1_CUT1_RppA (PF08392), Herpeto_peptide (PF14409), L_biotic_typeA (PF04604), LANC_like (PF05147), Lantibiotic_a (PF14867), MbtH (PF03621), NRPS (PF08415), Penicil_amidase (PF01804), Chal_sti_synt_C (PF02797), Chal_sti_synt_N (PF00195), Chalcone_3 (PF16036), PP-binding (PF00550), SchA_CurD (PF04486), Strep_pep (PF14404), TfuA (PF07812), Thioesterase (PF00975). The ketosynthase-alpha (KSa) domain from the polyketide synthase type II gene cluster HMMER profiles was from the RDP funcgene repository (Wang Q. et al., 2015). Resistance genes were also identified using HMMER and the Resfam database (Gibson et al., 2014). Full-length HMMER hits from the secondary metabolites were retrieved using esl-fetch. Taxonomy was assigned to the phosphopantetheine- (PP) binding domain of non-ribosomal polyketide synthase (NRPS) gene cluster and the KSa domain hits using GhostKOALA (Kanehisa et al., 2015).

## Results and Discussion

Nucleic acid extraction from soil samples resulted in an average of 17.9 ± 8.6 μg RNA per sample (range = 8.5–30.6 μg). Illumina sequencing yielded an average of 11,581,624 ± 4,804,223 reads per sample (range = 6,597,242–19,854,970). After MG-RAST quality control filters, an average of 83 ± 15% of reads were retained for further analysis (range = 59–97%). Of these sequences, an average of 25 ± 12% (range = 12–38%) were rRNA for each sample. Sequences of mRNA able to be annotated as protein-coding averaged 67 ± 14% (range = 46–83%) of reads per sample.

### Taxonomic Shifts

#### Domain-Level Changes

Based on taxonomic assignment of total RNA sequences, the domain *Bacteria* was dominant in all treatments (53, 82, and 82% of control, water, and OM samples, respectively), followed by *Eukaryota*, *Virus*, and *Archaea* (**Table 1**). The relative abundance of bacterial sequences increased with both water and OM treatments relative to controls, while eukaryotic abundances declined. Although present, archaeal sequences were rare in all treatments, ranging from 0.03 to 0.07% of the total sequences. Viral sequences were also detected in all treatments, ranging from <1 to 3% of total sequences.

**Table 1:** Percent abundances of sequences from taxonomic domains within treatments.

Treatment	Domain	Percent abundance
**Control**	Archaea Bacteria Eukaryota Virus Unclassified	0.08 52.82 37.99 1.45 7.66
**Water**	Archaea Bacteria Eukaryota Virus Unclassified	0.05 81.62 7.69 3.03 7.61
**OM**	Archaea Bacteria Eukaryota Virus Unclassified	0.04 82.45 10.62 0.89 6.00

#### Bacterial Phyla Responses

**FIGURE 2:**
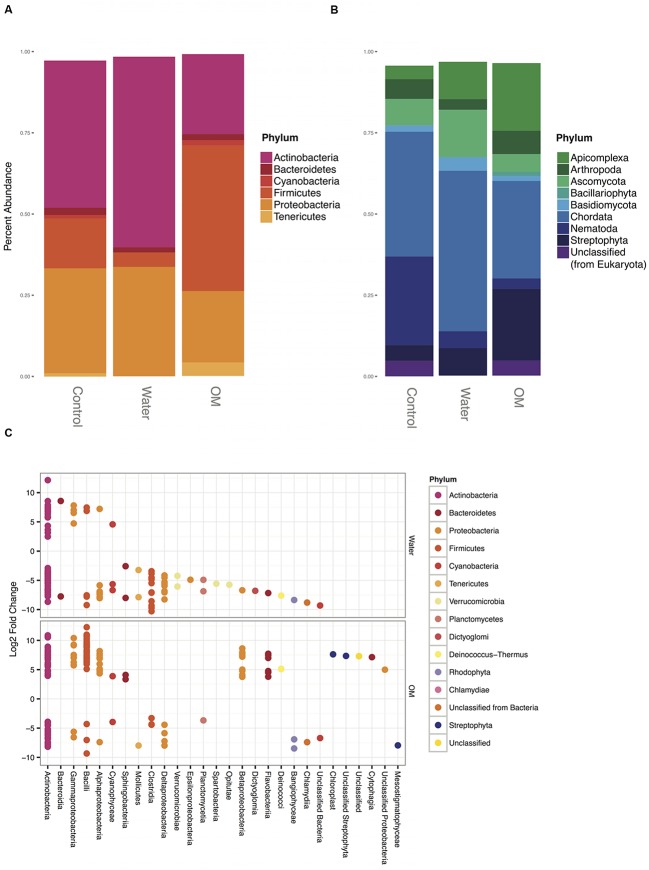
**Taxonomy of bacterial phyla (A) and eukaryotic phyla (B) representing >1% of sequences in at least two treatments, expressed as the percentage of the number of total sequences.** Differential abundance responses from the water and OM amendments versus the control **(C)**, expressed as the significant (*p*-adjusted value ≥0.05) log_2_ fold change of sequences in each treatment relative to the control, colored by phylum and grouped by class.

Bacterial sequences in all treatments were dominated by three phyla: *Actinobacteria*, *Firmicutes*, and *Proteobacteria* (**Figure 2A**). The phyla *Bacteroidetes*, *Cyanobacteria*, and *Tenericutes* were also present at >1% abundance, although *Cyanobacteria* and *Tenericutes* fell below that threshold in water treatments (**Figure 2A**). The mean dissimilarity of the bacterial communities across all treatments was 51% (SIMPER analysis, **Table 2**). Pairwise comparisons between treatments had a mean dissimilarity range of 72–78%. Five OTUs accounted for most of the community compositional differences between treatments, with all other OTUs contributing <5%. The top three OTUs that explained the most variance between each treatment pair were identified as *Actinobacteria* and *Firmicutes* in all comparisons. These OTUs were all more abundant in the water and OM treatments than in the control.

**Table 2:** Similarity percentage analysis (SIMPER) between treatments.

Control with water mean dissimilarity 72%						
**OTU**	**Control mean abundance**	**Water mean abundance**	**Mean dissimilarity**	**% Contribution**	**Cumulative %**	**Taxonomic assignment**

361352	26085	107286	18.83	26.31	26.31	Actinobacteria; Actinobacteria (class); Actinomycetales; Streptomycetaceae; *Streptomyces platensis*
361219	5073	22237	3.95	5.52	31.83	Actinobacteria; Actinobacteria (class); Actinomycetales; Streptomycetaceae; *Streptomyces lienomycini*
362477	2443	15667	2.57	3.60	35.43	Actinobacteria; Actinobacteria (class); Actinomycetales; Streptomycetaceae; *Streptomyces violaceolatus*

**Control with OM mean dissimilarity 78%**						

**OTU**	**Control mean abundance**	**OM mean abundance**	**Mean dissimilarity**	**% Contribution**	**Cumulative %**	**Taxonomic assignment**

51667	36	75736	6.75	8.65	8.65	Firmicutes; Bacilli; Lactobacillales; Carnobacteriaceae; *Carnobacterium* sp. AT7
361352	26085	33674	5.69	7.29	15.95	Actinobacteria; Actinobacteria (class); Actinomycetales; Streptomycetaceae; *Streptomyces platensis*
51664	13	47128	4.20	5.39	21.34	Firmicutes; Bacilli; Lactobacillales; Carnobacteriaceae; *Carnobacterium maltaromaticum*

**Water with OM mean dissimilarity 75%**						

**OTU**	**Water mean abundance**	**OM mean abundance**	**Mean dissimilarity**	**% Contribution**	**Cumulative %**	**Taxonomic assignment**

361352	107286	33674	14.52	19.30	19.30	Actinobacteria; Actinobacteria (class); Actinomycetales; Streptomycetaceae; *Streptomyces platensis*
51667	13.5	75736	6.47	8.60	27.91	Firmicutes; Bacilli; Lactobacillales; Carnobacteriaceae; *Carnobacterium* sp. AT7
51664	1.5	47128	4.03	5.36	33.27	Firmicutes; Bacilli; Lactobacillales; Carnobacteriaceae; *Carnobacterium maltaromaticum*

*Mean dissimilarity among samples = 51%*.

In general, water and OM amendments resulted in decreased abundances of bacterial phyla (**Figure 2C**). Members of only five phyla (*Actinobacteria*, *Bacteroidetes, Firmicutes, Proteobacteria*, and *Cyanobacteria*) had significant differential increases in abundance for water and OM treatments versus controls (**Figure 2C**). These phyla also had significantly increased functional transcripts (**Figure 4**). Because these phyla represent the strongest paired taxonomic and functional responses in this study, discussion of each phylum follows.

##### Actinobacteria

*Actinobacteria* are globally dominant in arid soils (Costello et al., 2009; Fierer et al., 2009; Pointing et al., 2009; Neilson et al., 2012). In MDV soils, DNA-based studies report declines in *Actinobacteria* in response to water and OM amendments of Schwartz et al. (2014) and Van Horn et al. (2014). In the present study, *Actinobacteria* produced the highest relative abundance of bacterial transcripts for the control (45%) and water addition (59%) samples, but declined (25%) in OM addition samples (**Figure 2A**). However, differential abundance analysis revealed varied responses of individual populations, with significant positive and negative log_2_ fold changes within the *Actinobacteria* class for both treatments (**Figure 2C**). Notably, the *Micrococcaceae* family had the strongest positive response of any *Actinobacteria* group in the OM samples and the third highest response (behind *Streptomycetaceae* and *Bifidobacteriaceae*) in the water samples of the present study, though the family had significant positive and negative differential abundance responses in both treatments. These strong positive responses are consistent with previous stable isotope work in these soils, that determined members of the *Micrococcaceae* family had the highest calculated growth rates of any bacterial group in water addition samples (Schwartz et al., 2014), suggesting that members of this family are poised to take advantage of transient water inputs in arid soils.

##### Bacteroidetes

In MDV soils, *Bacteroidetes* have been reported as one of the three most dominant phyla (Cary et al., 2010; Lee et al., 2012), using techniques that do not account for active versus inactive cells. In the present study, the relative abundance of *Bacteroidetes* was 2.3% of the control sample bacterial phyla, and declined in both water (1.6%) and OM (1.9%) samples. These results are consistent with a stable isotope study distinguishing active community members of these soils and amendments, which reported *Bacteroidetes* as a small portion of the bacterial community across all treatments (Schwartz et al., 2014).

Increased abundance of *Bacteroidetes* with OM enrichment has been observed in a variety of soils (Fierer et al., 2007, 2011), however, this phylum has shown inconsistent responses to OM amendments in MDV soils: relative abundance increased with algal mat leachate additions to soils (Van Horn et al., 2014), but decreased with mummified seal carcass (Tiao et al., 2012). In the present study, greater taxonomic resolution and differential abundance analysis provided a more detailed account of the taxonomic changes within this phylum. Members of three *Bacteroidetes* classes (*Cytophagia*, *Flavobacteriia*, and *Sphingobacteriia*) significantly increased in the OM treatments despite the overall decline of the relative abundance of the phylum.

##### Cyanobacteria

The phylum *Cyanobacteria* decreased in relative abundance in water treatments and increased in OM treatments (**Figure 2A**). However, the differential abundance of sequences within the phylum reveals a more complex pattern (**Figure 2C**). The orders *Nostocales* and *Chroococcales* made up the majority (>91%) of cyanobacterial sequences for all treatments and were the only orders contributing to cyanobacterial differential abundance responses across treatments. In both treatments *Nostocales* increased differential abundance (4.6 and 3.9 log_2_ fold change in the water and OM treatments, respectively) while *Chroococcales* decreased (-5.7 and -6.7 log_2_ fold change in water and -4.0 log_2_ fold change in OM).

*Cyanobacteria* are known inhabitants of soils and crusts in both hot and cold deserts, and are well adapted to the stresses of hydration and desiccation cycles, with response to wetting occurring within minutes (Garcia-Pichel and Pringault, 2001; Rajeev et al., 2013). Both *Nostocales* and *Chroococcales* are known inhabitants of harsh, cold environments, including MDV (Wood et al., 2008), barren high arctic (Elster et al., 1999; Kastovska et al., 2005), and high altitude Himalayan (Řeháková et al., 2011) soils. While both orders are known to thrive in subnival environments (Řeháková et al., 2011), *Nostocales* has been detected in greater abundance nearer the soil surface than *Chroococcales* (Elster et al., 1999). Resistance to disturbance or desiccation tolerance, factors that are correlated with soil depth, may contribute to the differing responses observed in the present study.

##### Firmicutes

*Firmicutes* was the third most abundant phylum in the control (15%) and water (4%) samples, but had the greatest increase in abundance in OM samples, contributing 45% of bacterial sequences in OM samples (**Figure 2A**). This striking increase by *Firmicutes* has been seen in other studies involving nutrient addition treatments to MDV soils, indicating that this phylum is adept at utilizing organic resources when available (Tiao et al., 2012; Schwartz et al., 2014; Van Horn et al., 2014). A high abundance of *Firmicutes* has also been reported in a metatranscriptomic study of Alaskan permafrost soils, highlighting the phylum’s ability to function in a frozen environment (Hultman et al., 2015). *Firmicutes* does not always increase in response to carbon-only amendments, and nitrogen amendments may also be necessary to trigger increases (Ramirez et al., 2010; Cederlund et al., 2014). Although only dissolved organic carbon was measured for the OM leachate added in the present study, the use of native algal mat as a complex nutrient source is presumed to have simultaneously increased nitrogen content in the soils.

##### Proteobacteria

*Proteobacteria* contributed 32% of control, 34% of water, and 22% of OM bacterial 16S rRNA gene sequences (**Figure 2A**). Previous DNA-based OM amendments studies in MDV soils have reported substantial relative abundance increases of only *Gammaproteobacteria* (Tiao et al., 2012), or of both *Beta-* and *Gammaproteobacteria* (Van Horn et al., 2014). The present study found that three classes (*Alpha-*, *Beta-*, and *Gammaproteobacteria*) had significant increases in abundance (**Figure 2C**), consistent with the findings of the only other study to distinguish active bacterial populations in OM amended MDV soils (Schwartz et al., 2014). Selectively sequencing active community members has increased detection of *Proteobacteria* in Alaskan soils as well, with a metatranscriptomic approach detecting greater abundances of *Proteobacteria* than either metagenomic or 16S rRNA gene based sequencing studies (Hultman et al., 2015).

##### Acidobacteria

Although *Acidobacteria* are a typical member of soil communities and have been reported in 16S rRNA gene studies of MDV soils (Lee et al., 2012; Van Horn et al., 2014), they represented only 0.04, 0.02, and 0.04% of bacterial sequences for control, water, and OM samples of this study, respectively, and were not significantly different in abundance for any treatment. This phylum was also not a dominant member of the active fraction of a stable isotope probing study of these soils, and it was detected in greater abundance in the inactive fraction of the same study (Schwartz et al., 2014). The *Acidobacteria* detected in 16S rRNA gene studies of these soils may be preserved non-viable cells, or simply slow or inefficient responders to increasing soil moisture and OM.

#### Eukaryotic Taxa

The domain *Eukaryota* contained 38% of sequences in the controls, and declined in relative abundance to 8% in water and 11% in OM amendments (**Table 1**). Discussion of key taxonomic groups and the challenges of eukaryotic annotation follow.

##### Chordata

Among eukaryotic phyla, a high proportion (38, 49, and 30% of control, water, and OM, respectively) of sequences were annotated as *Chordata* (**Figure 2B**), which was unexpected given the MDV are a microbially dominated ecosystem. The majority of reads annotated as *Chordata* fall into either the family *Muridae* (20, 31, and 74% of *Chordata* sequences in control, water, and OM treatments, respectively) or the family *Hominidae* (35, 36, 7% of control, water, and OM *Chordata*, respectively). While the MG-RAST pipeline uses Bowtie (Langmead et al., 2009) to filter out human sequences, reads with >60% identity and an alignment length of <35 bases were not culled from the dataset. As other studies have reported, contamination is typical in next-generation sequencing efforts to date and human reads are discovered in non-primate sequences across many databases (Malmström et al., 2005; Cibulskis et al., 2011; Longo et al., 2011; Kumar et al., 2013; Laurence et al., 2014). Additionally, there is bias toward intensively studied sequences, such as *Muridae*, in annotation databases (Wang et al., 2010), and unexpected mouse sequences have been detected in human sequencing projects (Robinson et al., 2010; Delviks-Frankenberry et al., 2012) and common DNA extraction and amplification reagents (Erlwein et al., 2011; Tuke et al., 2011). While *Chordata*-annotated sequences are included in all analyses for transparency, we do not speculate further on their presence or role *in situ*.

##### Arthropoda

The annotations within the phylum *Arthropoda* may also be skewed by model organism bias. The order *Diptera* made up the greatest percentage (39%) of *Arthropoda* sequences in the control samples. Only two endemic *Diptera* species have been reported on the Antarctic continent (Convey and Block, 1996), both from the midge family *Chironomidae*. However, the majority (51%) of control sample *Diptera* annotations were attributed to the fruit fly family *Drosophilidae* while *Chironomidae* annotations represented only 0.1%. Similarly, the common aquatic family *Daphniidae* comprised the majority of *Arthropoda* sequences in water (69%) and OM (75%) addition samples.

##### Nematoda

The eukaryotic phylum *Nematoda* was the next most abundant in control samples (27%), but declined in both water (5%) and OM (3%) addition samples (**Figure 2B**). Three nematode species are commonly reported in MDV soils and sediments: *Scottnema lindsayae*, *Eudorylaimus antarcticus*, and *Plectus antarcticus*, though none of these species are currently present in the M5NR database used to annotate this dataset. Rather, all differentially expressed functions linked to nematodes were attributed to the model organism genus *Caenorhabditis.* Dry MDV soils distant from streams or snowpack are dominated by a single species, *S. lindsayae* (Freckman and Virginia, 1998; Treonis et al., 1999; Gooseff et al., 2003). Abundance of *S. lindsayae* generally decreases with greater soil moisture, though this decline is not always statistically significant (Freckman and Virginia, 1997, 1998; Treonis et al., 1999; Gooseff et al., 2003). Additionally, there is no statistically significant relationship between nematode abundance and spatial distribution of soil OM in MDV soils (Freckman and Virginia, 1997; Gooseff et al., 2003). As the soils of this study were not adjacent to streams, the dry-adapted *S. lindsayae* was expected to be the dominant nematode species as predicted by a logistic regression habitat suitability model (Poage et al., 2008). Despite annotation resolution challenges, the relative abundance of *Nematoda* declined with both treatments that increased soil moisture in the present study, as would be expected for *S. lindsayae*.

##### Fungal phyla

In all samples, the two dominant fungal phyla were *Ascomycota* (8, 15, and 5% of eukaryotic sequences in control, water, and OM samples, respectively) and *Basidiomycota* (2, 4, and 2% in control, water, and OM, respectively) consistent with prior DNA-based survey of MDV soils (Fell et al., 2006). *Ascomycetes* have been reported as the dominant fungal group in a variety of soils across the continent (Lawley et al., 2004; Arenz and Blanchette, 2011), although in MDV soils with <5% soil moisture *Basidiomycota* were more widely-distributed (Fell et al., 2006). In the present study, relative abundances of both fungal phyla increased in water addition samples, consistent with prior positive correlations between fungal abundance and soil moisture in the MDV (Connell et al., 2006; Fell et al., 2006; Arenz and Blanchette, 2011). However, the relative abundances of both phyla decreased in OM addition samples, which is inconsistent with prior culture-based studies of Antarctic soils. In general, un-amended soil carbon concentrations are positively correlated with total fungal abundance (Arenz and Blanchette, 2011) and filamentous fungal abundance (Connell et al., 2006), and *Ascomycota* has been found to be an abundant decomposer in Antarctic soils where debris is present (Arenz and Blanchette, 2011). Fungal taxa are known to exhibit varied responses to carbon amendments in temperate soils based on specific substrate composition, and soil bacteria are known to outcompete fungi in some cases (Hanson et al., 2008; Chen et al., 2013). Varied results of fungal richness and diversity may occur due to sample size and number (Ranjard et al., 2003), as fungi are known to be spatially clustered (Foster, 1988; Horton and Bruns, 2001). Future amendment studies in MDV soils will require more robust sampling and additional analyses to better interpret fungal responses.

##### Streptophyta

The phylum *Streptophyta* contributed 5% of the control eukaryotic sequences (**Figure 2B**). The phylum was present in significantly greater relative abundance in the OM (22%) samples than control samples (**Figure 2C**). However, these sequences were exclusively chloroplast-derived annotations and interpretation of those results should be considered cautiously. Rather than indicative of plant species, the *Streptophyta* annotations may be considered a proxy for active photosynthetic organisms in these samples.

### Investigation of Transcript Functions

#### Transcript Annotation

**FIGURE 3:**
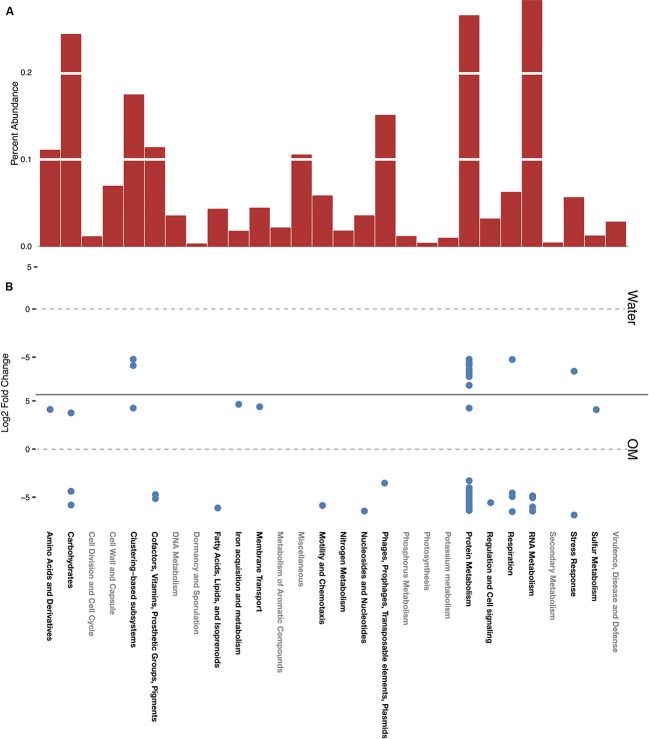
**Functional profile of the control, expressed as percent abundance of each subsystem (A).** All significant (*p*-adjusted value ≥0.05) differential expression for water and OM treatments, expressed as the log_2_ fold change of sequences in each treatment relative to the control **(B)**.

**FIGURE 4:**
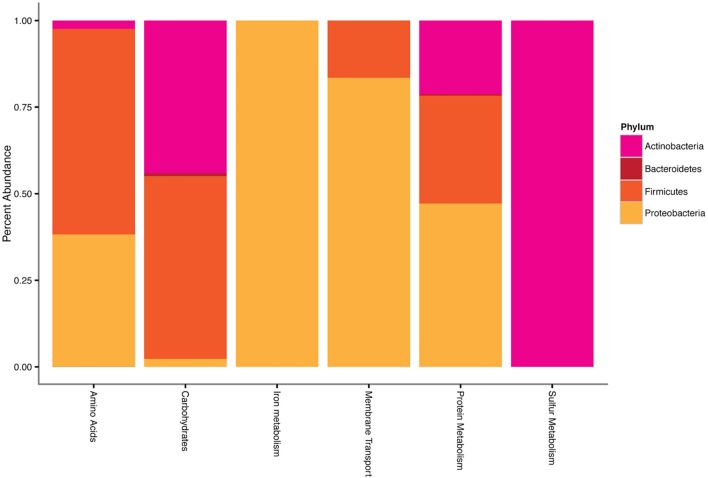
**Phylum-level taxonomy of sequences that had significant positive differential expression (log_2_ fold change), expressed as the percentage of total sequences for each differential expression result.** Phyla that contributed <1% of total sequences have been excluded.

**FIGURE 5:**
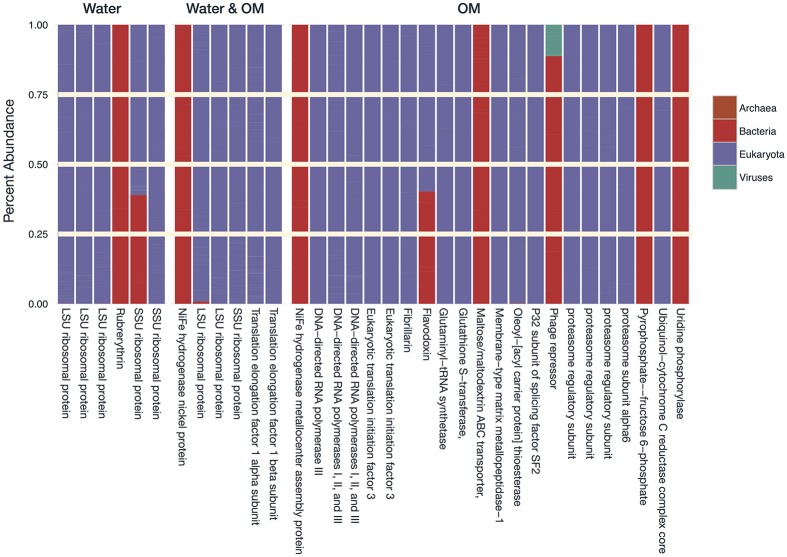
**Domain-level taxonomy of sequences that had significant negative differential expression (log_2_ fold change), expressed as the percentage of total sequences for each differential expression result.** Taxonomic assignments are based on the sequences in the control samples.

The SEED Subsystems functional annotations provide four levels of resolution, given as levels 1–4 in MG-RAST. Level 1, the highest level of functional categorization, referred to here as subsystems, is primarily considered, with further hierarchy described when relevant. A total of 28 subsystems were identified in the dataset. In control samples, representing the baseline functional profile of the soils in this study, the greatest relative abundance of sequences were in the subsystems of RNA metabolism, followed by protein metabolism, carbohydrates, and clustering-based (**Figure 3A**). RNA and protein metabolism subsystems contain many transcripts related to transcription, translation, and protein management and these subsystems have high representation in metatranscriptome studies (de Menezes et al., 2012), especially relative to metagenome studies (Urich et al., 2008). Annotations within the clustering-based subsystem (CBSS) are putative, based on the proximity of unknown sequences to those in a cluster of genes of known function (Gerdes et al., 2011). CBSS annotations were redundant, categorized into both CBSS and the putative function’s subsystem on the MG-RAST server. Thus, CBSS annotations are only shown in **Figure 3**, and redundant CBSS annotations are not presented in **Figures 4** and **5**.

#### Functional Profile of the Control Soils

The functional annotation of mRNA transcripts by the SEED Subsystems database revealed that the active portion of the MDV control soil microbial community is dominated by a chemoorganoheterotrophic bacterial community, but includes photoautotrophic cyanobacteria and algae, and various heterotrophic eukaryotes. Of particular note are the most abundant transcripts in carbohydrate and nitrogen metabolism subsystems, which reflect the community’s adaptation to the low OM content of these soils despite relatively high nitrogen and phosphorous content (Barrett et al., 2006). Complex carbon sources, such as plant cellulose material, are lacking in these soils, and simpler carbon sources are utilized (Barrett et al., 2005, 2006). Transcripts of the serine-glyoxylate cycle, a key component of methylotrophy (Anthony, 2011), comprised 11% of the carbohydrate subsystem, and maltose and maltodextrin utilization transcripts comprised another 11%. Photorespiration transcripts, a component of CO_2_ fixation, were less abundant (4%). Although organic nitrogen concentrations are low in these soils, inorganic nitrate concentrations are exceptionally high due to years of atmospheric deposition (Wada et al., 1981; Burkins et al., 2000; Barrett et al., 2002, 2007; Witherow et al., 2006). These nitrates appear to be valuable to the community as nitrate and nitrite ammonification transcripts were 31% of the nitrogen metabolism subsystem. Ammonia assimilation transcripts were also abundant (48% of nitrogen metabolism), but denitrification (6%) and nitrogen fixation (<1%) transcripts were rare.

Because *Actinobacteria* were a large portion of the control soil bacteria, we specifically searched for secondary metabolite genes (Amoutzias et al., 2008; Wang H. et al., 2014, 2015). The analysis focused on the PKSα domain of the PKSII gene cluster and the PCP domain of the NRPS gene cluster as these domains are highly conserved which make them good targets for analysis (Bachmann and Ravel, 2009). The majority of the transcripts detected were for proteins responsible for the assembly of polyketides (PKSII, 32% identified as ketoacyl synthase (KSα) genes) or non-ribosomal peptide synthetases (NRPS, 29% associated with the peptidyl carrier protein domain or PCP) which are known to produce a wide variety of secondary products. The taxonomic assignments of the KSα domain genes were largely from *Actinobacteria* (44%), but also *Proteobacteria* (27%) and *Firmicutes* (14%). The NRPS transcripts were similarly distributed among phyla *Actinobacteria* (50%), *Proteobacteria* (19%), and *Firmicutes* (16%). Resistance genes (including a range of anti-microbial compounds) were also considered, and the majority (84%) were for TEM beta-lactamase, a commonly occurring class of genes globally.

#### Differentially Expressed Transcripts

Transcripts of four subsystems were under-expressed in the water treatment relative to the control and transcripts of 14 subsystems were under-expressed in the OM treatment (**Figure 3B**). Significant over-expression was only detected in OM treatments: transcripts of seven subsystems were over-expressed (**Figure 3B**), but removal of redundant CBSS transcripts reduced the over-expressed subsystems to six (**Figure 4**). The over-expressed transcripts were predominately attributed to three bacterial phyla: *Actinobacteria*, *Proteobacteria*, and *Firmicutes* (**Figure 4**). Conversely, under-expressed transcripts were attributed largely to eukaryotic taxa in the control soils (**Figure 5**). Patterns within these results and their taxonomic designations are discussed below.

##### Carbohydrate utilization

All over- and under-expressed transcripts of the carbohydrate subsystem were identified as bacterial (**Figures 4** and **5**). The over-expressed transcript was that of 6-phosphogluconate dehydrogenase, decarboxylating (EC 1.1.1.44), which is the rate-limiting enzyme of the pentose phosphate pathway (PPP) that generates fructose-6-phosphate and reducing power. In addition, the PPP is responsible for generating metabolic intermediates for biosynthesis, catabolizing sugars that cannot be utilized by any other pathway (Sprenger, 1995), and NADPH, which may be used for mediating oxidative stress (Wang Y. P. et al., 2014). This transcript was over-expressed by several groups of bacteria: *Actinobacteria* (44%), *Bacteroidetes* (1%), *Cyanobacteria* (<1%), *Firmicutes* (53%), and *Proteobacteria* (2%; **Figure 4**). The *Proteobacteria* response was attributed to *Gammaproteobacteria* (98%) and *Deltaproteobacteria* (2%). The two under-expressed carbohydrate subsystem transcripts were of glycolysis and maltose/maltodextrin utilization pathways (**Figure 5**). *Actinobacteria* were solely responsible for the under-expressed glycolysis transcripts, while *Alpha*- (1%) and *Gammaproteobacteria* (30%), *Cyanobacteria* (<1%), and *Actinobacteria* (67%) all contributed to the under-expression of maltose/maltodextrin utilization. These functional shifts in the carbohydrate subsystem suggest that bacteria are responding to the carbon sources provided by the OM amendment.

Notably, *Actinobacteria* was the only phylum linked to each of the differentially expressed carbohydrate transcripts. This phylum has been linked with carbohydrate subsystem transcripts in other soils responding to disturbance events, including deforestation (Mendes et al., 2015) and inoculation after sterilization (Delmont et al., 2014). Additionally, all of the over-expressed sulfur metabolism transcripts were attributed to *Actinobacteria* (**Figure 4**). These transcripts were for the enzyme neuraminidase NanP, which is traditionally associated with pathogenicity but has been detected in three non-pathogenic genera of *Actinobacteria* in soils, and is thought to be used to metabolize sialic acids as a nutrient source in those cases (Gruteser et al., 2012). Sialic acid degrading enzymes have been observed previously in MDV soils, and were speculated to aid in degrading water-binding polymers of soil microorganisms (Gallikowski and Hirsch, 1988).

##### Transporters

All of the over-expressed transcripts in amino acids and membrane transport subsystems were of ABC-transport systems. Although a small portion (2%) was attributed to *Actinobacteria*, the phyla *Firmicutes* (59%) and *Proteobacteria* (38%) were largely responsible for over-expressed amino acid transcripts and were solely responsible for over-expressed membrane transport transcripts (**Figure 4**). The *Proteobacteria* response in amino acid transcripts was attributed to *Alpha-* (4%), *Beta-* (4%), and *Gammaproteobacteria* (91%), while that of membrane transport was attributed exclusively to *Gammaproteobacteria*. Transcripts in the amino acid subsystem were for polyamine transport, while those in the membrane transport subsystem were for oligopeptides. An increase in transport transcripts suggests increased nutrient availability and uptake potential, which is consistent with the complex OM addition of this study. Similarly, a comparison of frozen and thawed Alaskan soils also found higher abundances of transporter functions in thawed samples, which the authors deemed indicative of increased nutrient mobilization (Hultman et al., 2015).

##### Cellular Growth

All of the over-expressed protein metabolism transcripts were for protein biosynthesis, specifically bacterial SSU ribosomal protein S4p. Members of *Actinobacteria* (21%), *Bacteroidetes* (<1%), *Firmicutes* (31%), and *Proteobacteria* (47%) all contributed to the over-expression of these transcripts (**Figure 4**). As with the over-expressed amino acid transcripts, the *Proteobacterial* response was attributed to *Alpha*- (1%), *Beta*- (8%), and *Gammaproteobacteria* (91%). The increased expression of biosynthesis transcripts highlights that these bacteria were actively growing. Conversely, many of the under-expressed transcripts attributed to eukaryotic taxa were basic components of cellular growth, including ribosomal protein biosynthesis, polymerases, and translation factors (**Figure 5**). The over-expression of cellular growth transcripts in *Bacteria* and under-expression in *Eukaryota* likely attributed to the relative abundance shifts of these domains with treatments (**Table 1**).

##### Iron Acquisition

The over-expressed transcript in the iron metabolism subsystem (**Figure 4**) was for an enterobactin synthase component. Enterobactins are high-affinity iron-chelating compounds used for iron uptake, characteristically by the *Enterobacteriaceae* family (Fiedler et al., 2001). Indeed the over-expressed iron acquisition transcripts were attributed exclusively to that family of *Proteobacteria*.

## Conclusion

As part of the MDV coastal thaw zone (Marchant and Head, 2007; Fountain et al., 2014), the soils of this study are part of an “at risk landscape” in the Antarctic due to low elevation, proximity to the Ross Sea coast, permafrost ice abundance, and warm summer temperatures (Fountain et al., 2014). These soils are expected to experience increased water availability and mobilized nutrients due to thaw of permafrost and buried ice deposits (Fountain et al., 2014). The experimental amendments of the present study were designed to simulate expected climate change impacts, and they appear to be stressors in these soils, resulting in losses of taxonomic and functional diversity. The impending increases of soil moisture and OM due to climate change may diminish the taxonomic richness and functional capacity of these soil communities. The most significant positive responses were observed in the *Bacteria* (**Figures 2C** and **4**), whereas negative responses were largely among the *Eukaryota* (**Figure 5**). Many of the transcripts under-expressed by *Eukaryota* are indicative of basic cellular growth (**Figure 5**), thus repressed growth accounted for the observed taxonomic decline in abundance of *Eukaryota* in response to amendments (**Table 1**). Conversely, the transcripts lost by *Bacteria* were largely components of specialized pathways, rather than basic cellular functions (**Figure 5**). However, significant taxonomic losses occurred within the *Bacteria* as well. Three phyla (*Actinobacteria*, *Proteobacteria*, and *Firmicutes*) dominated the few positive transcript responses (**Figure 4**), and taxonomic diversity loss within *Bacteria* was apparent (**Figures 2A,C**). Dry soil bacterial communities of the MDV have been reported to contain distinct endemic taxa relative to wetted soils (Zeglin et al., 2011), and increased water and OM availability decreased diversity of dry soil bacterial communities (Schwartz et al., 2014; Van Horn et al., 2014). As climate change impacts the region, a loss of dry-adapted endemic oligotrophic taxa and dominance by generalist taxa is likely.

## Author Contributions

Experimental design by DV, JB, and CT-V. Project proposal and funding secured by DV, JB, MG, ES, and CT-V. Sample collection by DV. Sample processing by HB. Data analysis by HB and AW. Manuscript by HB, AW, CT-V, and DV.

## Conflict of Interest Statement

The authors declare that the research was conducted in the absence of any commercial or financial relationships that could be construed as a potential conflict of interest.

## Footnotes

1 http://metagenomics.anl.gov/linkin.cgi?project=12330
